# Cornual invasive hydatidiform mole: a rare case report and literature review

**DOI:** 10.1186/s12905-023-02727-z

**Published:** 2023-11-02

**Authors:** Jing Qian, Song Xu, Li Chen

**Affiliations:** 1 Department of Gynecology, Affiliated Hangzhou First People’s Hospital, Zhejiang University School of Medicine, No. 261 Huansha Road, Hangzhou, Zhejiang 310000 China; 2 Fourth Clinical School of Medicine, Zhejiang University of Chinese Medicine, Hangzhou, Zhejiang 310000 China

**Keywords:** Ectopic pregnancy, Chemotherapy, Gestational trophoblastic disease, Mole pregnancy, Invasive mole

## Abstract

**Background:**

The cornual pregnancy is a rare condition of ectopic pregnancies. Invasive hydatidiform mole is a rare form of gestational trophoblastic diseases. Cornual invasive hydatidiform mole is extremely rare.

**Case presentation:**

A 17-year-old girl presented to the gynecology department with irregular vaginal bleeding. This patient was diagnosed with cornual invasive hydatidiform mole. Mono-chemotherapy was admitted firstly and with poor efficacy. The patient was cured by a combination of chemotherapy and resection of the uterine mass.

**Conclusion:**

Cases with cornual invasive hydatidiform mole are extremely rare conditions. Unlike common site of invasive hydatidiform mole, mono-chemotherapy may be insufficient for cornual invasive hydatidiform mole. Chemotherapy in combination with other treatments may be needed in this rare condition.

## Background

The incidence of ectopic pregnancy is about 1.1%, and 95% of these ectopic pregnancies are tubal in origin [1]. The cornual pregnancy is a rare condition and represents 2–4% of ectopic pregnancies [2]. In general practice, practitioners used to use the term cornual pregnancy to refer to the gestations that occur near the uterine cornua, which is the junction of the oviduct and uterus [3]. Cornual pregnancy presents a complex diagnostic and management challenges, due to its inherent relationship to interstitial ectopic pregnancies. The incidence of complete hydatidiform mole is about 1–3 per 1,000 pregnancies [4] and malignant change occur in approximately 15 ~ 20% of complete hydatidiform moles [5]. Invasive mole is a subtype of malignant change of hydatidiform moles characterized by the presence of hydropic chorionic villi invading the myometrium with or without vascular and extrauterine invasion [6].

In this study, we present a case of a 17-year-old patient with ectopic invasive hydatidiform mole in the uterine horn. To the best of our knowledge, there are rarely cases with this particular condition. The clinical manifestations, microscopic characteristics, imaging features, diagnosis and treatment are discussed along with a literature review. Written informed consent was obtained from the patient to collect clinical data for publication.

## Case presentation

A 17-year-old girl, G4P1, who received an abortion in a local hospital with relatively outdated medical technology two months ago, presenting with irregular vaginal bleeding post-operation, was admitted to the gynecology department on April 1st, 2022. The patient did not have regular β-hCG monitor and gynecological ultrasound post abortion. Pathologists in our hospital borrowed and reviewed the pathological sections of the first curettage and the diagnosis of complete hydatidiform mole was confirmed.

**Fig. 1:**
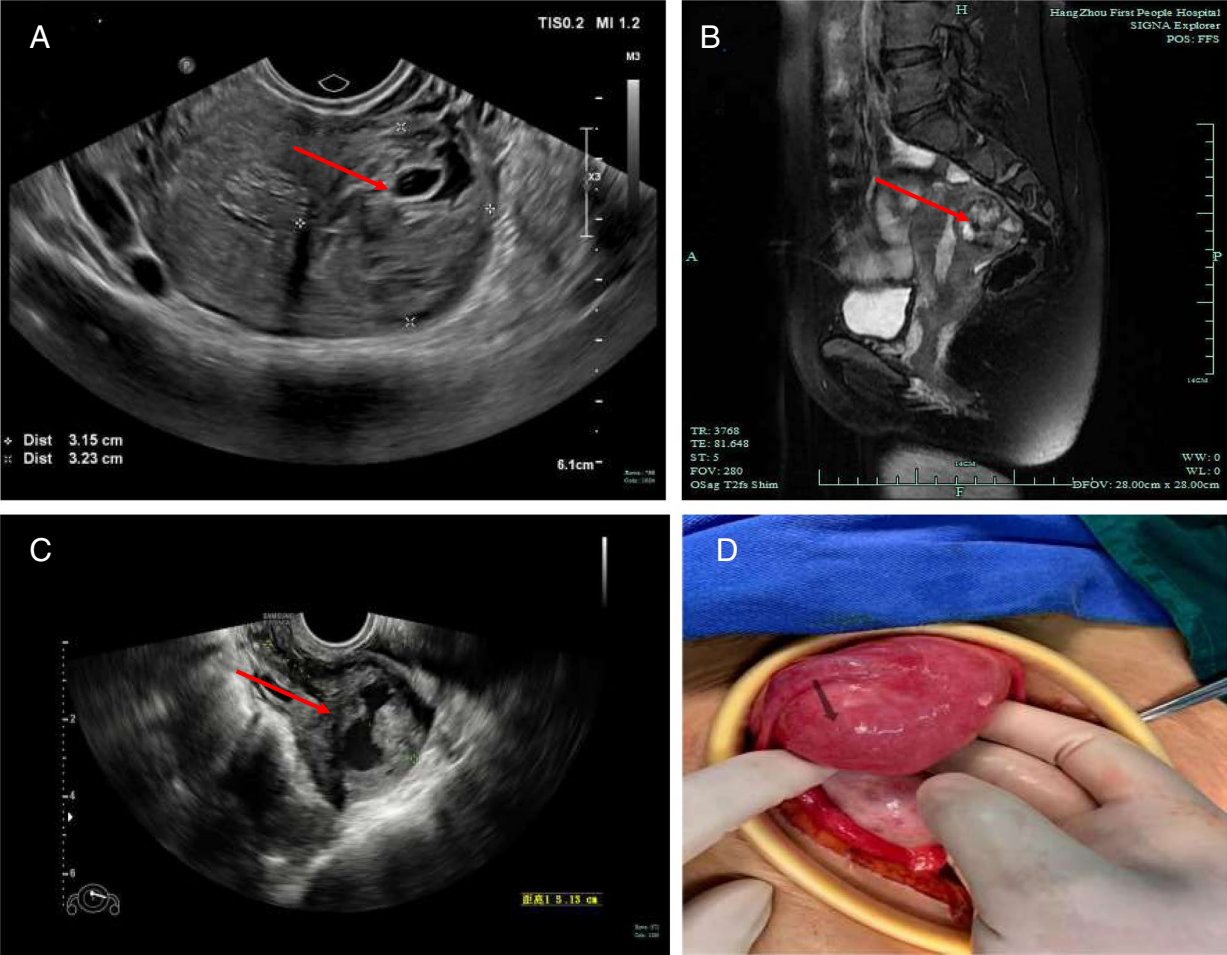
**A** Trans-vaginal ultrasound showed a left cornual heterogeneous echogenic mass about 4.3*3.2*3.2 cm. **B** Pelvis MRI scan showed a left cornual mass about 2.9*3.2*3.9 cm. **C** Trans-vaginal ultrasound showed a mixed-echo mass about 5.1*3.6*3.2 cm on the left uterine horn. **D** Open abdominal exploration revealed a round mass approximately 4 cm in diameter at the left uterine myometrium

β-hCG plasma level was 7,584 IU/L when she was admitted to our hospital and a trans-vaginal ultrasound found a heterogeneous echogenic mass (4.3*3.2*3.2 cm) with abundant blood flow signal in the myometrium of left uterine horn (Fig. 1A). A pelvic MRI scan was arranged and reported a left cornual mass (2.9*3.2*3.9 cm) likely representing gestational trophoblastic neoplasm (Fig. 1B). Gynecological examination, chest CT scan and other image examinations did not find metastasis. According to the anatomical staging system and prognostic scoring system developed by the Federation International of Gynecology and Obstetrics (FIGO) [7], the patient was diagnosed with gestational trophoblastic neoplasia (GTN) with a total score of 2 (1 point for pre-treatment serum β-hCG, 1 point for largest tumor size including uterus), stage I.

Systemic chemotherapy was commenced with methotrexate and folinic acid (methotrexate, 1 mg/kg intramuscular, every other day * 4 days, alternating with 15 mg of oral leucovorin* 4 days, repeated every 2 weeks). Although β-hCG plasma titer dropped significantly to 743.5 IU/L after the first course of chemotherapy, the patient still suffered from unexpected intermittent vaginal bleeding and her hemoglobin dropped to 55 g/L on May 5, 2022.

**Fig. 2:**
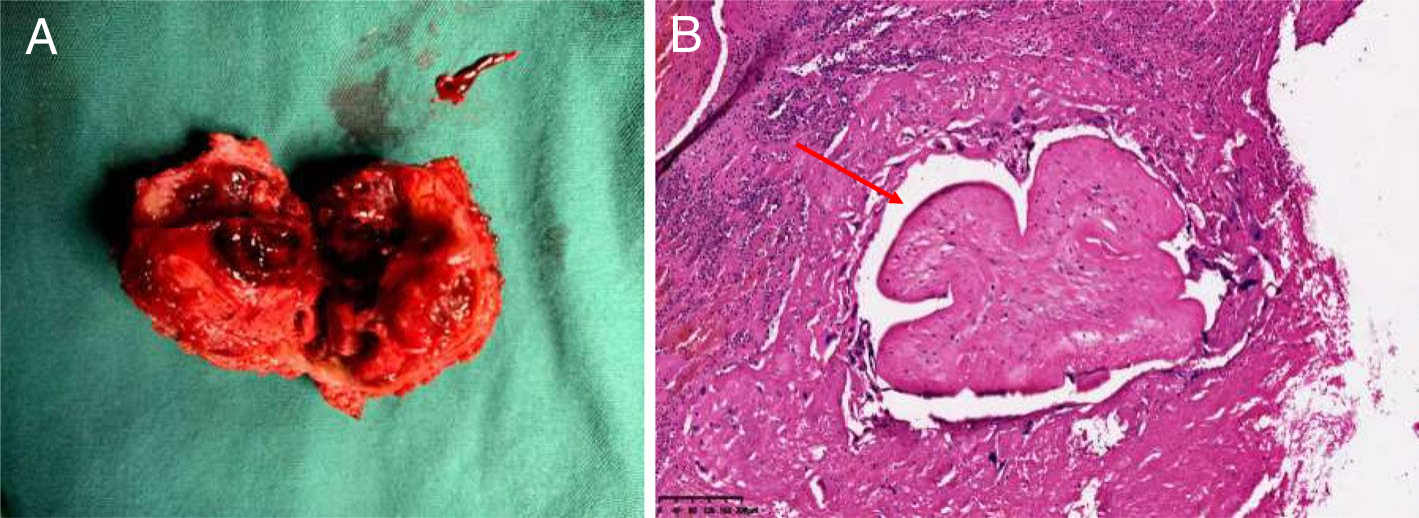
**A** Gross appearance of excised lesion. **B** Microscopic image of excised lesion. Arrow points to degenerated villus

Reexamined trans-vaginal ultrasound showed enlarged mixed-echo mass (5.1*3.6*3.2 cm) on the left uterine horn (Fig. 1C). In order to control vaginal bleeding and relieve anemia, we decided to perform an explorative laparotomy and resect the described uterine mass. Open abdominal exploration revealed a round mass approximately 4 cm in diameter at the left uterine myometrium (Fig. 1D). The lesion was excised by a wedge resection and the uterus was repaired by sutures to the deeper layer and serosa (Fig. 2A).

The microscopic examination (standard hematoxylin and eosin staining) of the surgical specimen showed smooth muscle with hemorrhagic, necrotic tissues and several degenerated villi (Fig. 2B). A diagnosis of invasive hydatidiform mole was made because of the presence of villus in the resected mass. β-hCG plasma titer fell to 51.2 IU /L after the operation. The patient continued to receive the original regimen of chemotherapy and the β-hCG level dropped to negative at the end of the third course of chemotherapy. Additional 2 courses of chemotherapy were given to prevent relapse. During the 8-month follow-up period, she had no evidence of recurrence with regular menstrual cycle.

## Discussion and conclusions

Cornual pregnancy is a rare form of ectopic pregnancy and only 2–4% of ectopic pregnancy developed in the cornual region of the uterus. Cornual pregnancy is one of the most hazardous types of ectopic pregnancy with a mortality rate of 2.0–2.5% [8]. Because of the abundant blood supply in the cornual region and thinning of the myometrium, severe hemorrhage and even death sometimes occurred from cornual rupture. Its management is poorly standardized and often guided by the clinical situation. Treatment requires evacuation of the pregnancy and hemostasis of the cornus if necessary [9]. Cornuotomy with suture and cornual resection with salpingectomy were proposed to achieve hemostasis of the uterine cornus [9].

Hydatidiform mole is a common form of gestational trophoblastic disease. The incidence of complete hydatidiform mole is about 1–3 per 1,000 pregnancies. Invasive hydatidiform mole may arise from molar pregnancy and the incidence is 1 per 15,000 pregnancies [10]. This case reports an extremely rare condition involving an invasive mole located in the uterine horn arising from complete hydatidiform mole. The pathogenesis of invasive mole arising from molar pregnancy remains unknown and etiologic risk factors that contribute to the development of invasive mole are unclear. Two previous reports have revealed a fascinating etiology, that is iatrogenic factors, such as uterine perforation and false passage, can lead to the transformation of invasive mole from molar tissues [11, 12]. The best treatment option for invasive mole is chemotherapy and the cure rate is nearly 100% in low-risk patients and 90% in high-risk patient [13].

Hydatidiform mole micmiking with ectopic pregnancy is a very rare situation. Less than 300 cases of ectopic molar pregnancy have been reported, among which 10% were in uterine horn [14]. Hwang JH et al. had ever reported a rare case of molar cornual ectopic pregnancy, which was successfully treated by the combination of laparoscopic cornuostomy and systemic methotrexate therapy [15]. Cornual invasive hydatidiform mole is extremely rare. Both Si MF et al.and Oskovi Kaplan ZA et al. had ever reported a case of invasive molar pregnancy in rudimentary uterine horn [16, 17]. The treatment of the two cases were all rudimentary horn resection. In our case, there was no congenital uterine anomaly. To the best of our knowledge, only one case with cornual invasive hydatidiform mole in the anatomically normal uterus has been reported [18]. Due to condition's rarity, the management of the cornual invasive hydatidiform mole is undefined and guided solely by the presenting condition. Whether mono-chemotherapy could be the only management option for the cornual invasive hydatidiform mole has yet to be confirmed by the future studies. In our case, although β-hCG plasma level dropped significantly after chemotherapy, abnormal uterine bleeding was not controlled and severe anemia appeared, which resulted in the decision of laparotomy. The abnormal uterine bleeding may be caused by the rich blood supply in the uterine horn and the high invasive ability of trophoblasts. The only reported invasive cornual mole case by Khlifi et al.also received a surgery (cornual resection with salpingectomy) and consecutively chemotherapy [18]. The two cases indicate that surgery may be required in cornual invasive mole in order to control vaginal bleeding and acute abdominal pain.

In conclusion, in this study, we report one of the rarest conditions: a patient with cornual invasive hydatidiform mole. We conclude through our case that mono-chemotherapy may not be the optimal management for this disease. Surgery combined with chemotherapy is needed in this rare condition.

## Data Availability

The datasets used and/or analysed during the current study are available from the corresponding author on reasonable request.
